# Posttraumatic Stress Symptoms Among Chinese College Students During the COVID-19 Pandemic: A Longitudinal Study

**DOI:** 10.3389/fpubh.2021.759379

**Published:** 2021-11-25

**Authors:** Xinli Chi, Liuyue Huang, Daniel L. Hall, Raissa Li, Kaixin Liang, Md Mahbub Hossain, Tianyou Guo

**Affiliations:** ^1^School of Psychology, Shenzhen University, Shenzhen, China; ^2^Health Policy Research Center, Mongan Institute, Massachusetts General Hospital, Boston, MA, United States; ^3^Department of Psychiatry, Harvard Medical School, Boston, MA, United States; ^4^Department of Health Promotion and Community Health Sciences, School of Public Health, Texas A&M University, College Station, TX, United States

**Keywords:** COVID-19, PTSS, longitudinal study, Chinese college students, descriptive survey study

## Abstract

A longitudinal assessment of the prevalence of posttraumatic stress symptoms (PTSS) and risk factors is indispensable for further prevention and/or treatment. The longitudinal web-based survey enrolled 1,164 college students in China. Measured at two time points (February and August 2020), PTSS, demographic information, adverse childhood experiences (ACEs), resilience and self-compassion information were collected to explicate the prevalence and predictors of PTSS concurrently and over time. Results showed that although PTSS generally declined throughout the 6 months after the outbreak of COVID-19, the prevalence remained relatively high. Resilience and self-compassion negatively predicted PTSS concurrently and longitudinally. While subjective family socioeconomic status (SES) and ACEs at Wave 1 did not predict PTSS under COVID-19 at Wave 1, but both significantly predicted PTSS at Wave 2. Findings implicate potential targets for detecting and intervening on symptoms of trauma in this vulnerable population.

## Introduction

Posttraumatic stress symptoms (PTSS) are commonly reported after experiencing or witnessing major stressful or distressing events. Its primary symptoms involve flashbacks of related memories, memories, avoidance of reminders of trauma and hypervigilance (1). Notably, in addition to direct exposure to life-threatening events, witnessing traumatic events may also cause PTSS (2). Existed studies of severe infectious respiratory diseases demonstrated that being threatened with infection or witnessing the death or serious injuries of others may conduce to PTSS (3–5). Indeed, the catastrophic consequences (e.g., a surge of critically ill patients, and the paralysis of medical systems) generated by the outbreak of COVID-19 were frequently reported by media (6), which were widely accessed by college students through the mass media, generating vicarious traumatization (7). In addition, due to the infectivity and high fatality rates of COVID-19, governments imposed shut-down measures. Students were confined to home with uncertainties and fear toward the future, which may result in mental health problems, including PTSS (8). Different from most studies on PTSS caused by one-off events, COVID-19-related PTSS is a global major public health event, which is found to be mutagenic and highly infectious, causing recurrent outbreaks which still affect people's lives. Furthermore, social media coverage of malignant consequences, such as sudden medical collapse, increases people's awareness of risk and may become chronic stressors.

Frequent and intense PTSS is a core criterion for the diagnosis of posttraumatic stress disorder (PTSD) (9). Furthermore, continually experiencing these symptoms results in weaker social functioning, lower quality of life, and a higher risk for self-harm and suicide (10). After 6 months of the SARS epidemic, the prevalence of PTSS was roughly 32% among the uninfected Chinese population and 55.1% among SARS patients (11). Likewise, the coronavirus disease 2019 (COVID-19), has affected many countries as a rapidly spreading infectious disease (12), inflicting substantial mental health problems (13). Studies on PTSS during COVID-19 have indicated that 23–37% of the general public worldwide are experiencing symptoms of PTSS (14, 15). A nationwide survey in China with more than 50 thousand participants found that the prevalence of COVID-related PTSS was 35% (16). Given the fact that COVID-19 may have long-lasting negative effects (17), longitudinal tracking of PTSS is essential as results can help policy-makers implement timely interventions for preventing high-risk behaviors and associated health outcomes.

To mitigate the psychological impacts of the COVID-19 pandemic, it is critical to clarify both the risk factors and protective factors associated with developing PTSS. According to the dynamic stress-vulnerability model (18), many factors can be identified as potential risk factors for the development and persistence of psychopathology (e.g., PTSS). The stress-vulnerability model is also known as the diathesis-stress model. The diathesis-stress model was firstly proposed by Meehl (19) to interpret the incidence of schizophrenia. After that, this model was widely used as to investigate other mental health problems (20, 21). As research has progressed, diathesis-stress factors have been broadened. No longer limited to biological factors only, multiple factors (e.g., psychological, biological, familial, and social) have also been included. It has prompted a more extensive and systematic examination of the determinants of specific psychiatric disorders. Likewise, the development of PTSS is not due to a single factor, but the result of a combination of multiple factors (22). Therefore, this study attempts to investigate the factors influencing the occurrences and changes of PTSS symptoms in college students using the dynamic stress-vulnerability model as the theoretical framework. These determinants mainly include demographic factors (e.g., age and gender), social vulnerability factors (e.g., socioeconomic status, family structure), and psychobiological vulnerability factors (e.g., adverse childhood experiences, high neuroticism, low resilience and self-compassion). These mentioned variables can be utilized as PTSS predictors among Chinese college students. Females may be more at risk than males for developing PTSS after traumatic events (23). With regard to age, one study found that older adults were more likely to develop post-earthquake PTSS (24).

As for social vulnerability, individuals from non-intact families were observed to be at higher risk for developing psychiatric disorders compared to those from intact families (25). A recent meta-analysis (*n* = 26,715 participants) examining the relationship between the family socioeconomic status (SES) and psychopathology found that individuals with low SES were more vulnerable to developing mental health problems (26). Chinese youths in rural areas also showed a higher prevalence of PTSS compared to their urban counterparts (27) which are the opposite of a study in Ireland during COVID-19 (3). However, research on the relationships between socio-demographic factors and PTSS under COVID-19 concurrently and over time remained limited.

Regarding psychological vulnerability, adverse childhood experiences (ACEs), as previous studies have shown, are positively correlated with higher PTSS (28). Individuals with childhood adversities are expected to be at greater risk for developing PTSS under COVID-19. However, longitudinal studies of ACEs on PTSS during the pandemic are limited and require further investigation.

Notably, self-compassion has been recognized as one of the most important positive factors in recent years. Self-compassion is the capacity to deliver love, kindness, and caring inward, particularly in distress (29, 30). Self-compassion involves being mindfully aware and kind toward oneself and viewing suffering as a larger part of the human experience following exposures to difficulties and hardships (31). High self-compassion is related to less maladaptive coping strategies (32), and presumably an emotion regulation strategy to cope with mental health problems (33). Individuals with high self-compassion tend to have fewer PTSS symptoms (34). Individuals with low self-compassion may be unable to down-regulate the brain's automatic survival response to the fight or flight response after major stressful events (35), resulting in psychological vulnerabilities. According to emotional processing theory (36), trauma information is often not processed appropriately due to avoidance of distress (e.g., triggering fear, which leads to emotional over-arousal). Notably, the level of self-compassion may be an important factor contributing to the development of the PTSS (34). Importantly, self-compassion promotes kind, mindful acceptance of negative emotions, which may be constructive to the gentle exposure of traumatic events as a way to reduce the corresponding PTSS symptoms. This portrays how establishing and maintaining a compassionate perspective may be conducive to developing healthy emotion regulation strategies that help alleviate the negative impacts of trauma.

In addition to self-compassion, resilience is another modifiable factor that attracted researchers' interest. Previous study has observed that individuals with low resilience demonstrated more psychological vulnerabilities to developing PTSS after major negative events (37). In comparison to self-compassion, the definition of resilience is more complex (38). One of the widely accepted definitions of resilience is the capacity to have hardiness, flexibility, and self-efficacy to adapt under stress (39–41). Individuals with high resilience may have an optimistic attitude toward adversities, helping them cope with negative events and reducing the risk of mental health issues (40). In contrast, low psychological resilience can contribute to more distress (42). Regarding potential psychological factors that may have an influence on PTSS, the emotional processing theory of PTSD suggested self-compassion may alleviate the negative influence of COVID-19 on PTSS. Furthermore, a considerable amount of studies showed the importance of resilience on the progression of PTSS (43, 44). Clinically, self-compassion and resilience are two important psychological variables which may serve as a way for the intervention of PTSS (45, 46). However, research on the longitudinal association between self-compassion, resilience, and COVID-19-related PTSS remains limited. Therefore, this study aims to examine which factors influence PTSS among college students in the context of the COVID-19 pandemic. In addition, to investigate the effect of psychological factors (in the present study resilience and self-compassion) on PTSS symptoms after controlling for demographic and familial factors.

Although previous studies of PTSS in China during stressful events including the COVID-19 outbreak provided valuable information, there are distinct research gaps that require scientific inquiries. Most of the available studies adopt a cross-sectional design, which cannot show the change in PTSS after stressful events such as the COVID-19 outbreak. Current longitudinal studies in China only cover a relatively short time frame (e.g., 1 or 2 months) (47, 48). Understanding the prevalence of PTSS during COVID-19 and its subsequent change longitudinally with a longer time period may promote more suitable approaches to address these problems. Most studies also focus on the mental health of the general public rather than PTSS in a specific population (e.g., college students). College is an important transition period from late adolescence to adulthood. During this period, young people are prone to encountering psychological crises that can involve feelings of insecurity, suspicion, and disappointment in life, sometimes referred to as a quarter life crisis (49). Thus, it is important to follow the mental health status of this group in a stressful situation such as COVID-19.

Thereby, based on the dynamic stress-vulnerability model and emotional processing theory, two waves of longitudinal data were collected over 6 months (February to August 2020) to develop the following study that has three main goals. The first goal is examining the incidence and variations of COVID-19-related PTSS among college students in China. The second is further investigating whether socio-demographic circumstances predict Chinese college students' PTSS under COVID-19 concurrently and over time. The third goal is to explore whether psychological variables (in this study, ACEs, self-compassion and resilience) are correlated with PTSS among Chinese college students concurrently and longitudinally.

## Methods

### Participants

Data for the present study was collected mid to late February in 2020 (Wave 1), ~1 month after the COVID-19 pandemic outbreak. After 6 months, the same subjects were called for the next round of survey in late August, 2020 (Wave 2). Participants were recruited from more than 100 colleges/universities across the country. All students who agreed to take part signed a consent form before filling out the questionnaires. In Wave 1, 1,218 students agreed to participate in the survey and 1,164 students eventually completed the questionnaire. The sample consisted of 410 (35.22%) males and 754 (64.78%) females (mean age = 20.56 years; SD = 1.90). From Wave 1 to Wave 2, 992 participants stayed in the study out of the original sample size. These participants were verified as the same participants from Wave 1 by matching their phone numbers with our original records. Further demographic information about the participants is summarized in Table 1.

**Table 1:** Comparisons of psychosocial variables and PTSS of the participants measured at Wave 1 by loss to follow-up.

**Variables**	**Follow-up**		**Loss to follow-up**		***p***
	**N/M**	**%/SD**	**N/M**	**%/SD**	
Age	20.48	1.80	20.64	1.99	0.17
Gender					0.82
Male	351	35.40	59	34.30	
Female	641	64.60	113	65.70	
Siblings					0.42
One child	317	32.00	60	34.90	
Non-one child	675	68.00	112	65.10	
Family intactness					0.56
Intactness	909	91.60	160	93.00	
Non-intactness	83	8.40	12	7.00	
SES	4.86	1.36	4.84	1.37	0.90
Residence					
Urban	557	56.10	90	52.32	0.59
Rural	435	43.90	82	47.68	
Resilience	35.19	6.20	36.41	5.71	0.44
Self-compassion	3.24	0.47	3.26	0.48	0.45
ACEs	1.33	1.79	1.27	1.64	0.48

*p obtained from Chi-square tests and t-tests*.

### Procedure

We recruited participants through an online survey. The invited students were asked to complete a questionnaire that included socio-demographic information and scales for subjective SES, self-compassion, adult attachment, and resiliency. After completing the questionnaire, participants received a compensation of ~10 RMB (~1.5 USD) which was provided online. There were no missing values due to the setup of the electronic questionnaire that required participants to answer each question. The project obtained ethical clearance from the Human Research Ethics Committee of the first author's affiliation.

### Instruments

#### Sociodemographic Characteristics

Participants were invited to report their age, gender (0 = male and 1 = female), family type (intact family or non-intact family), type of residence (urban areas = 0, rural areas = 1), and sibling status (no = 0, yes = 1).

#### Subjective Socioeconomic Status

The widely-used subjective SES scale was adopted to assess participants' subjective socioeconomic status (50). Participants were given a drawing of a ladder with 10 rungs and asked to choose a number that best represented their family's socioeconomic status.

#### PTSS

We revised the abbreviated PTSD Checklist-Civilian Version (PCL-C) to assess COVID-19-related PTSS. Item example: “*Repeated, disturbing memories, thoughts, or images of COVID-19 from the past?*” There are 6 items with 5 response options (from 1 = *not at all* to 5 = *extremely*). Scores range from 6 to 30, with higher scores indicating higher PTSS. As previous research studies characterized a score of at least 14 as an indication of PTSD (sensitivity 92%, specificity of 72%) (22). The Cronbach's α coefficient for the scale in this study was 0.81 in Wave 1 and 0.86 in Wave 2, respectively.

#### Self-Compassion

Participants were given the 26-item self-compassion scale (51) which assesses three aspects of self-compassion (negative aspects are reverse coded), including self-kindness, common humanity and mindfulness. Responses are identified on a 5-point scale from “*Almost Never*” to “*Almost Always*.” Higher scores indicate higher level of self-compassion. The questionnaire was validated in China previously (52). The Cronbach's α coefficient for the scale in this study was 0.87.

#### Resilience

The Abridged Connor-Davidson Resilience Scale is a self-administered questionnaire with a single dimension (53). It has 10 items with 5 response options (from 0 = *never* to 4 = *almost always*). The scale reflects the ability to tolerate stress and adversities. The final score is the sum of the responses for each item (range 0–40) where higher scores indicate higher resiliency. The scale's reliability was validated previously (54) and the Cronbach's α coefficient of the scale in this study was 0.92.

#### Adverse Childhood Experiences

Childhood trauma was assessed by the Chinese version of the Revised Adverse Childhood Experiences Scale (55). There were 14 items in the scale and were dichotomously coded (1 for presence and 0 for absence). Higher scores presented severer childhood adversities.

### Statistical Analyses

First, in order to check whether the loss of subjects had an impact on our results, we compared the data of the lost and retained subjects. Next, the percentages of participants who had PTSS based on screening procedures in the two waves were compared using related-samples McNemar tests to investigate the change in student PTSS after 6-months. Finally, the first hierarchical regression analysis was carried out to investigate the predictive effects of sociodemographic and psychological factors on PTSS concurrently. Specifically, PTSS was treated as a dependent variable, and demographic variables (i.e., age and gender) were considered independent variables, which were placed in the first step. After the controlled for age and gender, familial variables (i.e., family intactness, subjective SES, and residence) were placed in the second step. Thirdly, psychological factors (i.e., ACEs, resilience, and self-compassion) as independent variables were placed in the third step to assess their association with PTSS after controlled for variables in the first and second steps. Likewise, the second hierarchical regression was performed to explore their longitudinal relationships after the control for T1 PTSS in the first step. Statistical analyses were carried out using SPSS 23.0. The statistical significance was set at *p* < 0.05 (two-tailed) for the interpretation of the results.

## Results

### Comparisons of Followed-Up Participants and Attrition

14.78% (*n* = 172) of the baseline participants did not complete the study in Wave 2 (Table 1). Differences were not statistically significant between the retained and dropped participants. Of the retained participants, the mean age was 20.48 years old (SD = 1.80). Thirty-five point four percent of participants were male and 68.00% reported having sibling(s). Approximately 8.40% of students reported coming from non-intact families and ~56.10% of participants were from urban areas.

### Screening for PTSS

As can be seen in Table 2, 30.80% of the college students in Wave 1 were identified as having PTSS while 27.40% of students in Wave 2 were identified as having PTSS 6 months later. These data suggest that the COVID-19 related PTSS among China's college students is generally high.

**Table 2:** Prevalence and change of PTSS in Waves 1 and 2 (February 2020 and August 2020).

**PTSS total score**		**PTSS (Cut-off ≥ 14)**		
Wave 1 (M/SD)	Wave 2 (M/SD)	Wave 1 (%)	Wave 2 (%)	*x* ^2^
11.79 ± 4.25	11.38 ± 4.27	30.80	27.40	3.35^†^

† *p = 0.06*.

### Differences of PTSS at Two Time Points

We performed related-samples McNemar tests to compare PTSS levels at two time points. The results show that the change was borderline significant. Participants showed lower PTSS levels at Wave 2 then at Wave 1 (*x*^2^ = 3.35, *p* = 0.06) (Table 2).

### Correlates and *T*-Test Results of PTSS in College Students

In the bivariate correlation analysis, the following variables were correlated with PTSS in Wave 1: age, ACEs, resilience and self-compassion (*ps* < 0.05). Independent *t*-tests showed significant differences in PTSS between rural and urban areas (*t* = 2.11, *p* < 0.05). There was marginal significance between intact and non-intact families (*t* = −1.89, *p* = 0.059). In Wave 2, gender, SES, ACEs, resilience and self-compassion were correlated with PTSS (*ps* < 0.05) (Table 3).

**Table 3:** Correlations or *t*-test results of independent variables with PTSS in Waves 1 and 2 (February 2020 and August 2020).

	**Age**	**Gender**	**Siblings**	**Family intactness**	**SES**	**Residence**	**ACEs**	**Resilience**	**Self-compassion**
PTSD-Wave 1	0.09^**^	−1.75	1.26	−1.89^†^	0.01	2.11^*^	0.12^***^	−0.31^***^	−0.32^***^
PTSD-Wave 2	0.00	−2.05^*^	1.10	−0.98	−0.06^*^	0.07	0.19^***^	−0.31^***^	−0.31^***^

† *p = 0.059*.

* *p <0.05*;

** *p <0.01*;

*** *p <0.001*.

### Predictors of PTSS in College Students

The results of hierarchical regression analyses were shown in Table 4. In Wave 1, age positively predicted PTSS as older participants manifested more severe PTSS. Gender could not predict PTSS in both waves (β = 0.06, β = 0.04, *p* > 0.05). Residence also positively predicted PTSS as participants in urban areas reported higher PTSS. Resilience and self-compassion significantly predicted PTSD concurrently and longitudinally at 6 months as low amounts of both psychological variables were associated with higher levels of PTSS concurrently (β = −0.17, *p* < 0.001; β = −0.22, *p* < 0.001) and longitudinally (β = −0.12, *p* < 0.001; β = −0.08, *p* = 0.021). Subjective SES did not predict PTSS under COVID-19 in Wave 1. However, it significantly predicted participants' PTSS in Wave 2 (β = −0.07, *p* = 0.021). Likewise, ACEs did not correlate with PTSS in Wave 1 but they significantly predicted PTSS in Wave 2 (β = 0.12, *p* < 0.001).

**Table 4:** Multiple linear regression of predictors of PTSS in Waves 1 and 2 (February 2020 and August 2020).

**Dependent variable: PTSS-Wave 1**						**Dependent variable: PTSS-Wave 2**					
**Independent variables**	**B**	**SE**	**β**	***t***	**Δ*R*^**2**^**	**Independent variables**	**B**	**SE**	**β**	***t***	**Δ*R*^**2**^**
						**Step 1**					0.22^***^
						*PTSD-Wave 1*	0.50	0.03	0.47	16.69^***^	
**Step 1**					0.01^***^	**Step 2**					0.00
Demographic factors						Demographic factors					
Age	0.20	0.07	0.09	2.85^**^		Age	−0.08	0.07	−0.04	−1.25	
Gender	0.52	0.27	0.06	1.94		Gender	0.33	0.25	0.04	1.30	
**Step 2**					0.01	**Step 3**					0.01*
Familial factors						Familial factors					
Family intactness	0.75	0.46	0.05	1.63		Family intactness	0.06	0.43	0.00	0.14	
Subjective SES	−0.01	0.10	0.00	−0.06		Subjective SES	−0.21	0.09	−0.07	−2.32^*^	
Residence	−0.61	0.27	−0.08	−2.31^*^		Residence	0.25	0.25	0.03	1.01	
**Step 3**					0.12^***^	**Step 4**					0.04^***^
Psychological factors						Psychological factors					
ACEs	0.1	0.08	0.05	1.45		ACEs	0.30	0.08	0.12	3.86^***^	
Resilience	−0.11	0.02	−0.17	−4.52^***^		Resilience	−0.08	0.02	−0.12	−3.38^**^	
Self-compassion	−1.92	0.32	−0.22	−5.92^***^		Self-compassion	−0.75	0.32	−0.08	−2.32^*^	

* *p <0.05*;

** *p <0.01*;

*** *p <0.001*.

## Discussion

This study showed the prevalence of PTSS under COVID-19 among Chinese college students with an interval of 6 months. In addition, the concurrent and longitudinal predictive effects of demographic, familial, and psychological factors on PTSS were examined. 30.8% of Chinese college students reported having PTSS in February but this number went down to 27.4% in August, 2020. The prevalence of PTSS among college students in China under the COVID-19 pandemic was lower than that after the Wenchuan earthquake among trauma-affected people (45.5%) (56). However, the prevalence of PTSS was almost as high as that of the SARS outbreak (31.18%) among the public in epidemic-affected areas (11). This may be because COVID-19 occurred over a longer time period in contrast to the Wenchuan earthquake which was sudden. Individuals are aware that their risk of being infected by COVID-19 can be reduced if the correct precautions are taken, which may lead to a less sense of losing control, resulting in a less severe posttraumatic stress reaction (57). Of note, despite the absence of a large-scale outbreak of COVID-19 in provinces other than Hubei provinces (worst-hit area), people in other regions may also be severely affected by the outbreak of COVID-19. The results are in consonance with some existed research on major stress events (58, 59). Potential reasons for this include the “amplification” effect of risk events (58, 59). Specifically, residents of light and non-affected areas primarily resort to the media to obtain COVID-19-related information. This dissemination through the media or other informal pathways may affect an individual's risk perception and thus influences PTSS symptoms.

The prevalence rates of COVID-19-related PTSS in the present study were similar to two studies on the general Italian population (age range: 18–64, and 18–89, respectively), with a prevalence rate of 27.7–35.6% (60, 61). These were close to the prevalence rate of 31.8% in the third study with American young adults (aged from 18 to 30) during COVID-19 (62). Nevertheless, other research studies on similar samples depict a lower incidence of PTSS. For instance, Karatzias et al. found that 17.7% of adults (age: 18 to more than 65) in Ireland, and 16.79% in the United Kingdom reported COVID-19-related PTSS (3). One possible explanation of the disparity may be different screening tools adopted in the investigations, with some tools having higher cut-off points. In addition, the severity and development stage of the pandemic locally may also affect PTSS. Future studies can advance the research of PTSS during COVID-19 with representative samples, longitudinal studies, or meta-analysis.

Wave 1 of the study found that age and residence significantly predicted PTSS during COVID-19 in college students. Higher PTSS was reported with increasing age. A possible reason for this may be that older students may be experiencing more stress due to the difficulty of finding employment during the uncertainty of COVID-19 (63). Moreover, the results from the hierarchical regression showed that gender did not predict PTSS in this study, which is inconsistent with previous research (64). This may be due to the fact that, unlike certain traumas, infectious diseases such as COVID-19 are threatening regardless of gender. In addition, type of residence was associated with PTSS in Wave 1 which indicated that college students in urban areas experienced more PTSS compared to their rural counterparts. This may be due to the fact that the outbreak of COVID-19 during February, 2020 in China spread more rapidly in major cities with greater population densities. However, age and residence did not predict PTSS in Wave 2. This could be because Wave 2 data was gathered in late August, 2020 when COVID-19 was better controlled in China, compared to the situation in February, 2020. Consequently, older students could have more positive outlooks on their employment opportunities and urban residents may no longer fear the rapid spread of the virus in their cities.

Surprisingly, we found that individuals with fewer ACEs and higher SES reported lower levels of PTSS in Wave 2 while the differences were not significant in Wave 1. One possible explanation for this is that at the beginning of the pandemic, all individuals, regardless of their ACEs and SES, may be affected concurrently by the acute stress of the public health emergency. However, as the pandemic progressed and the economy deteriorated, those with higher family affluence have more resources to buffer stress from the economic downturn compared to individuals with low SES (65). Individuals with more ACEs may also have less effective strategies for emotion regulation, making them more vulnerable to negative adjustment longitudinally (66). As a result, despite COVID-19 infection numbers decreasing drastically by Wave 2 in August, 2020, the economic and psychological effects from the virus are long-lasting and likely still deeply impact individuals with higher ACEs and low SES.

We further found that self-compassion was significantly associated with PTSS concurrently and longitudinally as lower levels of self-compassion predicted higher levels of PTSS. These findings are similar to several studies conducted in America (67, 68). Self-compassion emphasizes kindness toward one's self and mindful awareness of distressing experiences which activates the mammalian caring system (69). This neurocognitive mechanism may lead to fewer posttraumatic stress symptoms. In addition, individuals with high self-compassion may be better at self-regulating their stress levels and adapting coping strategies such as constructively reframing or accepting difficulties (70), resulting in less severe PTSS. These findings suggest that individuals with low self-compassion are more psychologically vulnerable and have more risk factors, putting them at greater risk for being psychologically affected by the COVID-19 pandemic.

We also found that greater resiliency negatively predicted PTSS which is in line with previous study (71). One possible reason is that individuals with high resiliency are more adaptive when facing difficulties, enabling them to recover from negative events (72). High levels of resilience can help individuals suffering from trauma recover more quickly (73). As previous research has found, resilience can lead to positive emotions (74) as it can enhance happiness and promote psychological health which buffers negative psychological effects caused by major stressful events. Our results suggest that elevating levels of resiliency can diminish PTSS.

Several limitations should be noted in this study. First, our participants were mainly from Guangdong, Anhui, Hebei, and Jiangsu provinces in China. Survey data from the strongly-affected province (Hubei) accounted for a very small proportion (1.57%); thus, the findings may not be generalizable across the Hubei province. Furthermore, the present study assessed COVID-related PTSS. Therefore, generalizing the results to PTSS to other major stressors demands caution. Given this study mainly focused on the concurrent and longitudinal association between several demographic, social, and psychological variables on PTSS under the COVID-19 pandemic, the interactions between variables on PTSS were not examined. Future studies can explore their relationships and the underlying mechanism. Moreover, although the Abbreviated PCL checklist showed good psychometric properties in previous studies, the pandemic caused widespread suffering, and the reports from mass media exacerbated vicarious trauma. Given that we did not assess the fear and threat severity of COVID-19 among participants, the results may not preclude the possibility of false positives. It is preferable to assess the stress intensity of COVID-19 for each individual in future studies to improve data accuracy. Lastly, the general methodological limitations of self-reported surveys should be considered, which may affect the interpretations of the measured constructs as well as the generalizability of the study's findings. Further studies should be conducted using different methods of data collection to collect objective data (e.g., clinician-rated or bioindicators), minimize methodological biases, and explore potential psycho-pathological mechanisms that may complement such advanced investigations.

Despite these limitations, this study has several results in aiding our understanding of PTSS among college students during the COVID-19 pandemic. The study can likely be generalized to most Chinese college students as it is based on a relatively large sample size. The incidence of PTSS among college students in China is relatively high, suggesting that policy-makers, educators, and clinical professionals need to take timely and effective measures to reduce the PTSS of college students thus promoting their healthy development. We found that individuals with low SES and ACEs longitudinally were more prone to develop PTSS under COVID-19, which suggests an immediate need for mental health interventions for this vulnerable population. Additionally, factors such as resilience and self-compassion may be protective factors against the negative effects of stressful events on mental health of college students at a single time point and over time. Recently, there have been calls for synchronous and asynchronous remote delivery of resiliency interventions to address COVID-19 stress (75). Our findings suggest that such programs can be particularly helpful if targeted to college students with histories of trauma or low SES backgrounds. Therefore, prospective measures for improving resiliency and self-compassion in college students may be a way to mitigate the impact of the COVID-19 pandemic on mental health and a strategy to improve general well-being in the future.

## Data Availability Statement

The raw data supporting the conclusions of this article will be made available by the authors, without undue reservation.

## Ethics Statement

The studies involving human participants were reviewed and approved by the project obtained ethical clearance from the Human Research Ethics Committee of the Shenzhen University (No: 2020005). The patients/participants provided their written informed consent to participate in this study.

## Author Contributions

XC, LH, and TG took a principal role in designing the study, writing the protocol, developing methodologies, and drafting the manuscript. LH performed the statistical analysis. DH, RL, KL, and MH edited the manuscript. All authors have read and approved the final manuscript.

## Funding

TG was supported by National Natural Science Foundation of China (31871115). XC was supported by Guangdong Basic and Applied Basic Research Foundation (2021A1515011330).

## Conflict of Interest

The authors declare that the research was conducted in the absence of any commercial or financial relationships that could be construed as a potential conflict of interest.

## Publisher's Note

All claims expressed in this article are solely those of the authors and do not necessarily represent those of their affiliated organizations, or those of the publisher, the editors and the reviewers. Any product that may be evaluated in this article, or claim that may be made by its manufacturer, is not guaranteed or endorsed by the publisher.
